# Town and Country

**Published:** 1859-06

**Authors:** 


### TOWN AND COUNTRY.

PASSING down Broadway at high noon of a midsummer's day, the sign of the "Polar Refrigerator," brings with it associations most delightfully refreshing. We think at once of eternal snows and arctic ice, and before we come to ourselves are peering inside, in imagination ; and the mouth waters at the rich yellow grass butter and the farm house milk not ten hours old of the Rockland company, their buttermilk and their cream; with dishes piled heaping high, with luscious strawberries; why this is nothing short of living in the country, with its inconveniences left out; its noonday rides in scorching suns and dusty roads; its morning walks through dripping grass; its worms on your pillow, its bugs in your bed; to say nothing of the hours from eleven till four, spent in sweltering and panting under roofs all guiltless of a single sha e tree. In fact, boarding at "farm houses" is only in name; the reality is in New York City, in any house within gun shot of Union Square, where the breezes come fresh from the sea, right across from the bosom of the broad Atlantic, only a dozen miles away. It is not the most pleasant thing in the world to exchange the cool and roomy and clean apartments of your own dwelling, for a single, so-so room, of a "farm house," in which single room, entire families must pass a large part of the twenty-four hours, the principal occupation being in surmises as to what will be for the next meal; what is the reason that "splendid" cows never give any fresh milk ; that magnicent hens never lay any fresh eggs; and that fresh butter and spring chickens are never seen on the table of the fine old "farm house." The fact is a "farm house" within fifty miles of a large city, is no farm house at all; it is a country tavern, no more, no less ; its doors are open to Tom, Dick and Harry; "first come, first served," and where decent quiet people are made to make acquaintance with every body, with Miss Jemima Smith, the brewer's daughter, and John Jones, the rich distiller's heir, and various other "people," who, when you come to town, intrude themselves upon you by virtue of a boarding-house acquaintance, and refuse to be shaken off. A cut askew and a cut direct are no cuts at all. They are so tremendously forbearing, that even a kick is apologized for as a "mistake." They praise you up to every body, as "friends of ours." They leave cards an acre broad, and a whole salver full in no time. And although you are never "at home," as to them, they do not take it amiss, but construe it to the extensiveness of your acquaintance, which leaves you but little time for any thing else but visiting them. Every card they leave is an occasion for "bringing you in" to those of their own circle, by remarking "the last time I called on Mr. and Mrs. Prince, of the Avenue, they were not in, which I much regretted, as I esteem them highly, and have known them for some time."

To children and young people, spending the summer in the country may be made highly advantageous; but it is questionable whether those who have passed forty-five, are not better off in their own homes in the city, enjoying their undisturbed routine, and the quiet comfort which attaches to sameness at the change to the down hill of life. To such, an excursion for a day or two has its advantages; but beyond that, it is for the most part, ordinarily, a penance and a bore, unless, in the few cases where a "home" in town, can be exchanged for a "home" in the country.

